# Health Impacts of Nursing Home Staffing

**DOI:** 10.1001/jamahealthforum.2025.6272

**Published:** 2026-01-16

**Authors:** Andrew Olenski, Karen Shen, Krista Ruffini, Ashvin Gandhi

**Affiliations:** 1 Department of Economics, Lehigh University, Bethlehem, Pennsylvania; 2 Bloomberg School of Public Health, Johns Hopkins University, Baltimore, Maryland; 3 McCourt School of Public Policy, Georgetown University, Washington, DC; 4 Anderson School of Management, University of California, Los Angeles

## Abstract

This case-control study examines mortality, hospitalization, and emergency department visits among nursing home residents after an Illinois payment reform.

## Introduction

Adequate staffing is critical to the effective operation of health care facilities. This is particularly true in nursing homes, where the Centers for Medicare & Medicaid Services considers staffing to have “the greatest impact on the quality of care nursing homes deliver.”^1^ Yet, most nursing homes staff below the levels that clinicians, researchers, and advocates consider adequate.^2^ Various state and federal proposals have aimed to raise staffing through payment reforms and staffing regulations, including the Centers for Medicare & Medicaid Services’ recent attempt to set more stringent staffing standards.^3^

To understand the impacts of such policies on patient health, we study a recent Illinois payment reform aimed at increasing staffing at Medicaid-serving facilities. This policy incentivized greater staffing by providing facilities with bonus Medicaid reimbursements up to $38.68 per Medicaid resident-day based on acuity-adjusted staffing levels. In previous work, we found that this reform increased nurse staffing by 12.2% relative to baseline.^4^ Herein, we examine the reform’s impact on patient health.

## Methods

We used linked Medicare claims and Minimum Data Set assessments from the second quarter of 2021 to the third quarter of 2023 to examine mortality, hospitalization, and emergency department visits among nursing home residents. As nursing home care aims to promote and aid residents’ independence in activities of daily living (ADLs), we also examined ADL scores, with lower values implying greater independence. Because the policy primarily targeted Medicaid-focused facilities serving long-stay patients, we restricted the sample to quarterly and annual health assessments for long-term residents (N = 2 558 611).

We estimated 2 difference-in-differences models comparing changes in health outcomes before and after the reform for patients in treated Illinois facilities (ie, those with above-median Medicaid shares prior to the reform) against 2 distinct control groups: (1) high-Medicaid facilities in other states (across-state analysis) and (2) low-Medicaid facilities in Illinois (within-state analysis). We present event study and pooled difference-in-differences estimates from these models. All estimates adjust for patient health and demographic characteristics (eMethods in Supplement 1 provides additional details).

This study was approved by the National Bureau of Economic Research institutional review board, which waived need for participant consent. This study followed the STROBE reporting guidelines.

## Results

**Figure 1.**
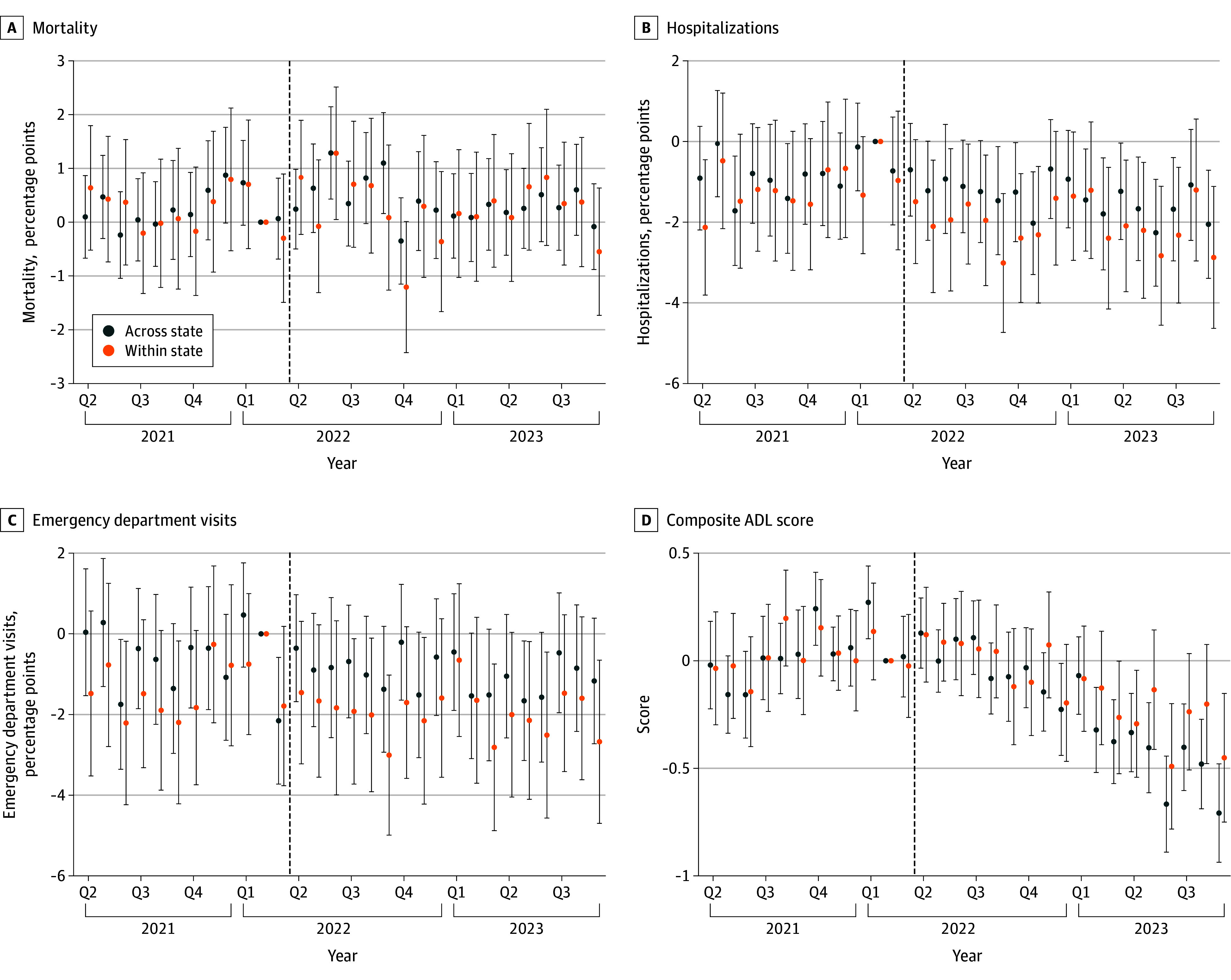
Event-Study Estimates for Impact of Reform on Claims-Based and Activities of Daily Living Health Outcomes This figure presents estimates for event study regressions over long-stay (quarterly [Q]/annual) assessments. Across-state comparisons show patterns in Illinois to other states among high-Medicaid facilities. Within-state comparisons show patterns in high-Medicaid facilities to low-Medicaid facilities in Illinois only. Each point reflects an estimate for a calendar month, with February 2022 as the omitted period. Vertical bars denote 95% CIs with standard errors clustered by facility. Dashed lines denote the treatment date. Higher activities of daily living (ADL) scores indicate worse health, so negative values indicate beneficial effects.

Figure 1 depicts the event study estimates resulting from both the across-state and within-state analyses. The patterns do not suggest violation of the requisite parallel trends assumption, supporting the validity of both control groups as counterfactuals for Medicaid-focused nursing homes in Illinois.

**Figure 2.**
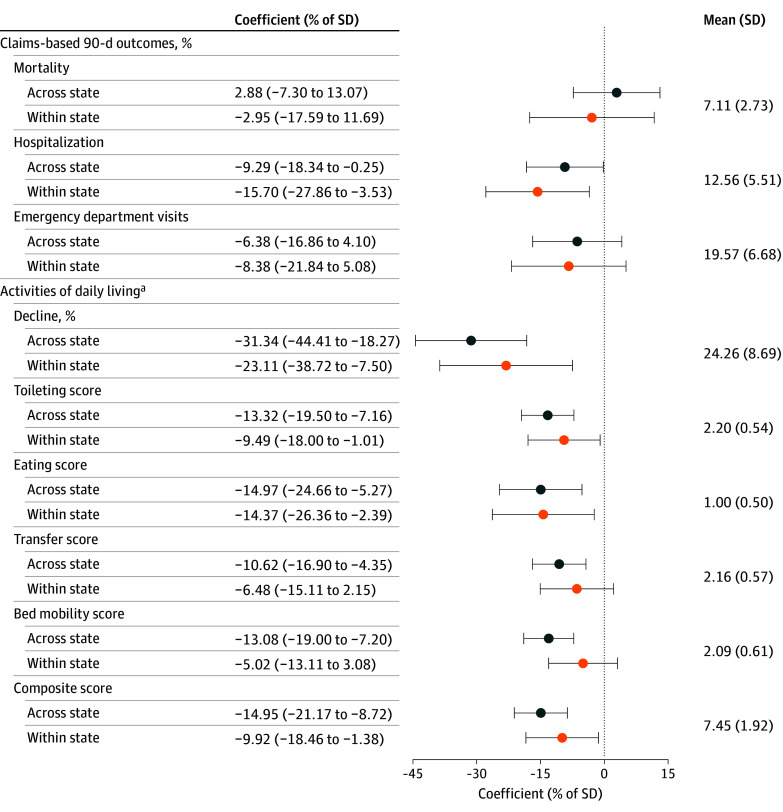
Difference-in-Differences Estimates for Impact of Reform on Claims-Based and Activities of Daily Living Health Outcomes This figure presents estimates from difference-in-differences regressions of multiple health measures for long-stay patients. Results omit a donut hole of one quarter on each side of the treatment date (April 1, 2022). The across-state sample includes facilities with a facility-level Medicaid share exceeding the Illinois median in 2019; the within-state sample includes all facilities in Illinois. Negative values indicate beneficial health effects. Horizontal bars denote 95% CIs, with standard errors clustered by facility. Means and SDs are calculated across facilities, after averaging each health outcome at the facility level for all long-stay patients in the sample period. Figure 1 presents corresponding event studies. ^a^Measures that became financially incentivized during the analysis period.

Figure 2 summarizes the estimates of the health impacts of the reform. Both analyses find some evidence that the induced staffing increases improved patient health. Using the across-state estimates after the reform, 90-day hospitalization rates decreased by 0.51 percentage points (95% CI, −1.01 to −0.01 percentage points) for patients in Illinois relative to patients in other states, which was a reduction of approximately 4.1% of the mean. The within-state estimates were broadly similar.

Both analyses found substantial improvements in independence in ADLs. However, these changes in ADLs should be interpreted cautiously: because bonus payments were based on acuity-adjusted staffing levels, facilities could increase their payments by reporting residents as low acuity and requiring little ADL-related care. It is therefore possible that reductions in ADLs reflect strategic downcoding rather than real health improvements.

## Discussion

In this case-control study, we found that a Medicaid policy that incentivized high staffing levels was associated with modest improvement in some dimensions of patient health. However, even modest effects are extremely meaningful at scale: these estimates suggest that if a similar reform were adopted nationally, there would be 6142 fewer hospitalizations each year.

While these findings are promising, they leave considerable room for further research. The financial incentive structure in this setting prevents us from distinguishing whether improvements to ADLs and other financially tied factors are truly health improvements rather than downcoding. Future work examining other staffing incentives may address this. Additionally, many of these estimates lack precision, which can be improved by studies of larger quasi-experiments.

## Notes

Supplement 1.
eMethods

eReference

Supplement 2.
Data Sharing Statement
