# Supplementary material for: Health Impacts of Nursing Home Staffing
Source: JAMA Health Forum. 2026 Jan 16;7(1):e256272. doi: 10.1001/jamahealthforum.2025.6272 (PMC12811805; doi:10.1001/jamahealthforum.2025.6272)
Supplement: Supplement 1. — eMethods eReference [file jamahealthforum-e256272-s001.pdf]

Supplemental Online Content

Olenski A, Shen K, Ruffini K, Gandhi A. Health impacts of nursing home staffing. *JAMA Health Forum*. 2026;7(1):e256272. doi:10.1001/jamahealthforum.2025.6272

eMethods
eReference

This supplemental material has been provided by the authors to give readers additional information about their work.

© 2026 Olenski A et al. *JAMA Health Forum*.

A Additional Information on Data and Methods

A.1 Details on Staffing-Level Reimbursement Reform

More than 60% of nursing home bed-days are paid for by Medicaid. Medicaid reimbursement formulas vary across states, but typically provide a per-diem amount for each Medicaid resident, with possible adjustments for factors such as geography or care needs.

On April 7, 2022, the Illinois legislature passed House Bill 0246 (HB0246), which aimed to increase staffing by changing how the state's Medicaid program reimbursed nursing facilities.[1] The largest component of HB0246 was a staffing level incentive payment for nursing homes. These incentive payments provided an additional $9 per Medicaid-resident-day for facilities achieving 70% of the clinical target and gradually increased to a maximum of $38.68 per Medicaid-resident-day for facilities achieving at least 125% of the target. Crucially, the incentive payments were large compared to the base per-diem, which averaged $181.78 before the reform. Additionally, only 11.0% of Illinois facilities staffed above 125% prior to the reform, indicating that the upper threshold was rarely binding.

A facility's "target" staffing level was calculated based on the care needs of the residents of the facility. To measure resident care needs, the federal government uses mandatory health assessments to categorize each resident into one of 66 Resource Utilization Groups ("RUGs") based on the severity of their clinical and functional needs measured on a number of dimensions including ADLs, cognitive performance, and mood. CMS then maps these RUGs to a "clinical target" amount of care based on the CMS Staff Time Resource Intensity Verification (STRIVE) study. Summing the STRIVE targets of patients in a facility gives a case-mix-adjusted clinical target level of staffing for the facility as a whole. The ratio of a facility's actual staffing level to its target staffing level is known as the "STRIVE ratio," and was the measure used in the Illinois staffing reform.

A few caveats are important to note regarding the reform. First, many researchers and advocates believe that the STRIVE targets are substantially lower than the number of hours needed to provide high-quality care, and therefore the ideal staffing level exceeds a 100% ratio.[1] Second, STRIVE ratios used for the Illinois incentive payments did not distinguish between the hours of care provided by staff with differing levels of certification (i.e. RNs vs. LPNs vs. CNAs). Finally, because the policy only increases Medicaid reimbursements, payments for residents whose stays were being paid for by Medicare or private insurance were not affected by this reform. Thus, facilities with more Medicaid residents are more "exposed" to the payment reform and should therefore be more responsive. Our analyses therefore focus on "high-Medicaid" facilities; that is, facilities that had above-the-Illinois-median share of residents covered by Medicaid in 2019 (58.3%). This information comes from facility-level Medicaid utilization provided in the LTCFocus database.[2]

A.2 Study Sample, Data Sources, and Methods

Our study population consists of long-stay patients in U.S. nursing homes from 2021Q2-2023Q3. Each CMS-certified facility is required to conduct routine assessments on all of their residents on at least a quarterly basis and submit this information to CMS. This assessment data, termed the

[1]Though the bill was formally signed into law on May 2022, we treat April 1 (i.e. the start of 2022Q2) as the "event date" because executive approval was virtually certain given that the governor supported the bill. In addition, due to a phase-in of the staffing incentive payments, April 1, 2022 was the start of the first quarter in which the full staffing incentive schedule was in effect; we thus refer to this date as the "effective" date of the reform.

[2]LTCFocus is sponsored by the National Institute on Aging (1P01AG027296) through a cooperative agreement with the Brown University School of Public Health.

© 2026 Olenski A et al. *JAMA Health Forum.*

Minimum Dataset (MDS), provides information on numerous patient health measures. Our analysis examines regularly-scheduled (quarterly and annual) assessments.

In addition to the MDS data, we also examine detailed Medicare claims and enrollment data. Medicare Provider Analysis and Review (MedPAR) files were used to identify hospitalizations. We identify emergency department visits using a combination of the MedPAR, inpatient, and outpatient claims data. We measure mortality using the date of death provided in the Medicare Beneficiary Summary Files. The remaining health outcomes are measured in the MDS data. Note that our enrollment and claims-based outcomes include one observation per MDS assessment and are measured based on occurrence (e.g., hospitalization or death) within 90 days after that assessment. In doing so, our claims-based analysis maintains the same structure as our assessment-based analysis.

We implement two difference-in-differences (DD) models. One approach compares facilities in Illinois that served a high share of Medicaid residents to facilities in other states that served a similarly high share of Medicaid residents ("across-state"). The second approach compares high-Medicaid Illinois facilities to facilities in Illinois that served a lower share of Medicaid residents ("within-state") Each approach includes month and facility fixed effects, and interaction terms between dummy variables denoting assessments conducted in Illinois high-Medicaid facilities and a "post" indicator for the period after the policy was in place. We exclude a "donut hole" of one quarter on each side of the treatment date (April 1, 2022) to reduce the influence of anticipation and adjustment periods. This difference-in-differences approach is valid under the assumption that without the payment reform, high-Medicaid Illinois facilities would have followed similar trends in each health outcome as each control group.

All analyses include set of patient controls. We control for patient demographics (sex, race, and age) in addition to the DRG code of the most recent hospitalization preceding each assessment (or an indicator for having no observed prior hospitalization). Due to computational constraints, the across-state sample consists of 100% of Illinois residents and a 50% random sample of residents in all control states. The within-state sample consists of all Illinois residents.

The 95% confidence intervals around all reported estimates reflect an alpha level of 0.025 in each tail, and have not been adjusted for multiple comparisons. All standard errors are clustered at the nursing home level. All analyses were performed with the use of Stata software, version 18.5 (StataCorp), and R, version 4.4.2.

A.3 "Incentivized" Health Measures and Detail on Additional Components of the Reform

In this section, we discuss how two sets of coding incentives changed during our sample period. The first set of coding incentives are due to the normalization used in the staffing level payment reform. The second set of coding incentives are due to Illinois shifting the acuity measures used in its base Medicaid payment for nursing homes. We detail these below.

Downcoding incentive due to staffing payment reform (STRIVE) Recall from Section A.1 that the staffing payment incentive compensated facilities based on their staffing levels normalized by the acuity of their patients. This normalization is intended to ensure that incentive payments were directed to facilities whose staffing levels were high relative to the clinical need of their patient population. The intent was that holding the acuity of a facility's residents fixed, the facility would receive larger payments by increasing staffing levels. However, a potential unintended consequence of this design was that a facility could also change the size of its staffing payments by holding staffing levels fixed and reporting its residents as low-acuity and therefore requiring less intensive care. Acuity was calculated according to the Staff Time Resource Intensity Verification

© 2026 Olenski A et al. *JAMA Health Forum.*

(STRIVE) study, which as noted in Section A.1, calculates an expected number of hours of care for each of 66 acuity groupings from the RUG-IV grouping. For example, the RUG-IV system takes into account the ADL needs of the residents, so that the STRIVE expected hours are higher for RUG groups with higher ADL needs. Thus, as a result of the staffing incentive reform, nursing homes may have been incentivized to "downcode" measures of patient acuity that were included in the RUG-IV grouping system.

Incentives due to change in base rate (PDPM) The STRIVE-based payments we study were made in addition to base Medicaid per-diem rates. Illinois, like many states, pays larger base per-diem rates to facilities with higher acuity patients.

In addition to the staffing reform, Illinois was also one of the first states to switch its Medicaid base payment from utilizing acuity measures from RUG to acuity measures from the Patient Driven Payment Model (PDPM). Illinois phased in the change from October 1, 2022 to September 30, 2023, by gradually increasing the share of the base Medicaid per-diem that relied on PDPM and reducing the share that relied on RUG.

Since base rates are higher for facilities with higher acuity patients, facilities are incentivized to upcode acuity according to the measurement system being used. As Illinois shifted to using PDPM, this strengthened the incentive to upcode measures that contribute to PDPM-acuity and reduced the incentive to upcode on measures that contribute to RUG-acuity. One such measure were ADL measures, which were used in the RUG system but not the PDPM system. Insofar as measures contribute to both RUG and PDPM, the effect is more ambiguous.

© 2026 Olenski A et al. *JAMA Health Forum*.

eReference

[1] Charlene Harrington, Mary Ellen Dellefield, Elizabeth Halifax, Mary Louise Fleming, and Debra Bakerjian. Appropriate nurse staffing levels for us nursing homes. *Health Services Insights*, 13, 2020.

© 2026 Olenski A et al. *JAMA Health Forum*.
