# Supplementary material for: Health Impacts of Nursing Home Staffing
Source: JAMA Health Forum. 2026 Jan 16;7(1):e256272. doi: 10.1001/jamahealthforum.2025.6272 (PMC12811805; doi:10.1001/jamahealthforum.2025.6272)
Supplement: Supplement 2. — Data Sharing Statement [file jamahealthforum-e256272-s002.pdf]

Data Sharing Statement

Olenski. Health Impacts of Nursing Home Staffing. *JAMA Health Forum*. Published January 16, 2026. doi:10.1001/jamahealthforum.2025.6272

Data
Data available: No

Additional Information
Explanation for why data not available: Confidential claims data
